# Pharmacologically blocking p53-dependent apoptosis protects intestinal stem cells and mice from radiation

**DOI:** 10.1038/srep08566

**Published:** 2015-04-10

**Authors:** Xinwei Wang, Liang Wei, Julie M. Cramer, Brian J. Leibowitz, Colleen Judge, Michael Epperly, Joel Greenberger, Fengchao Wang, Linheng Li, Matthias G. Stelzner, James C. Y. Dunn, Martin G. Martin, Eric Lagasse, Lin Zhang, Jian Yu

**Affiliations:** 1 Department of Pathology, University of Pittsburgh School of Medicine, 5117 Centre Avenue, Pittsburgh, PA 15213; 2 University of Pittsburgh Cancer Institute, 5117 Centre Avenue, Pittsburgh, PA 15213; 3 Department of Radiation Oncology, University of Pittsburgh School of Medicine, 5117 Centre Avenue, Pittsburgh, PA 15213; 4 Department of Pathology, University of Kansas Medical Center, Stowers Institute for Medical Research, 1000 E 50th Street, Kansas City, MS 64110; 5 Department of Surgery, Veterans Affairs Greater Los Angeles Healthcare System, Los Angeles, CA 90073; 6 Departments of Surgery and Pediatrics, David Geffen School of Medicine, University of California, 10833 Le Conte Ave, Los Angeles, CA 90095; 7 Department of Pharmacology and Chemical Biology, University of Pittsburgh School of Medicine, 5117 Centre Avenue, Pittsburgh, PA 15213

## Abstract

Exposure to high levels of ionizing radiation (IR) leads to debilitating and dose-limiting gastrointestinal (GI) toxicity. Using three-dimensional mouse crypt culture, we demonstrated that p53 target PUMA mediates radiation-induced apoptosis via a cell-intrinsic mechanism, and identified the GSK-3 inhibitor CHIR99021 as a potent radioprotector. CHIR99021 treatment improved Lgr5+ cell survival and crypt regeneration after radiation in culture and mice. CHIR99021 treatment specifically blocked apoptosis and PUMA induction and K120 acetylation of p53 mediated by acetyl-transferase Tip60, while it had no effect on p53 stabilization, phosphorylation or p21 induction. CHIR99021 also protected human intestinal cultures from radiation by PUMA but not p21 suppression. These results demonstrate that p53 posttranslational modifications play a key role in the pathological and apoptotic response of the intestinal stem cells to radiation and can be targeted pharmacologically.

Radiation therapy continues to serve as a leading cancer treatment. However, exposure to high levels of ionizing radiation (IR) leads to acute gastrointestinal (GI) injury and development of GI syndrome^1^. This toxicity is the most significant dose-limiting factor in abdominal radiotherapy, and there is currently no FDA-approved agent for its prevention or treatment^2^,^3^,^4^. The intestinal epithelium undergoes rapid and continuous renewal fueled by the intestinal stem cells (ISCs) located at the bottom of crypts, including fast cycling crypt base columnar cells (CBCs) and more quiescent “+4” cells above Paneth cells (PCs) in mice^5^,^6^,^7^. Lgr5 can mark both cells, while Bmi1 and HopX were reported to preferentially mark +4 cells^7^. Loss of clonogenic or stem cells plays a key role in radiation-induced acute intestinal injury and lethality^1^, and is regulated by the p53 pathway and its transcriptional targets PUMA and p21^4^,^8^,^9^,^10^. PUMA-dependent apoptosis rapidly depletes ISCs and progenitors in hours following high dose radiation, and *PUMA* deficiency results in enhanced animal survival and crypt regeneration via p21-dependent DNA repair^11^,^12^.

Glycogen synthase kinase 3 (GSK-3) is an essential serine/threonine protein kinase consisting of two isoforms, GSK-3α and GSK-3β, that regulates a wide variety of cellular functions such as metabolism, proliferation and survival^13^,^14^. Numerous GSK-3 inhibitors have been developed and used in functional studies, including non-selective Lithium, and more selective small molecules such as SB216763, SB415286 and CHIR99021^14^,^15^. The regulation of GSK-3β is complex and highly tissue-specific, and it has both pro- and anti-apoptotic functions^15^. For example, GSK-3β is required for normal development and inhibits the canonical Wnt pathway by promoting the degradation of β-catenin^15^. GSK-3β can promote apoptosis in response to DNA damage in neurons^16^,^17^,^18^, and growth factor-mediated activation of PI3K/AKT phosphorylates and inhibits GSK-3β^19^. p53 is extensively modified after DNA damage^20^, and K120 acetylation of p53 is linked to induction of PUMA and apoptosis after radiation and mediated by GSK-3-dependent phosphorylation of Tip60 at S86 in some cancer cells^21^,^22^,^23^. The role of GSK-3 in the DNA damage response of intestinal stem cells remained undefined.

Radiation-induced intestinal injury and protection has traditionally been studied in mice^4^. In the present study, using a three-dimensional (3D) *in vitro* intestinal crypt culture system^24^, we demonstrated a cell-intrinsic role of p53 and PUMA-dependent apoptosis in radiation-induced intestinal injury, and identified the GSK-3 inhibitor CHIR99021 as a potent intestinal radiation protector. Our findings in mouse and human intestinal cultures and whole mouse, indicated that CHIR99021 treatment strongly protects Lgr5+ ISCs by selectively inhibiting p53-dependent induction of PUMA and apoptosis through p53 posttranslational modification not protein level. We believe this is the first comprehensive study to date modeling radiation-induced ISC injury and protection using crypt culture.

## Results

### *PUMA* deficiency strongly protects intestinal crypts and Lgr5+ cells from radiation in culture

**Figure 1:**
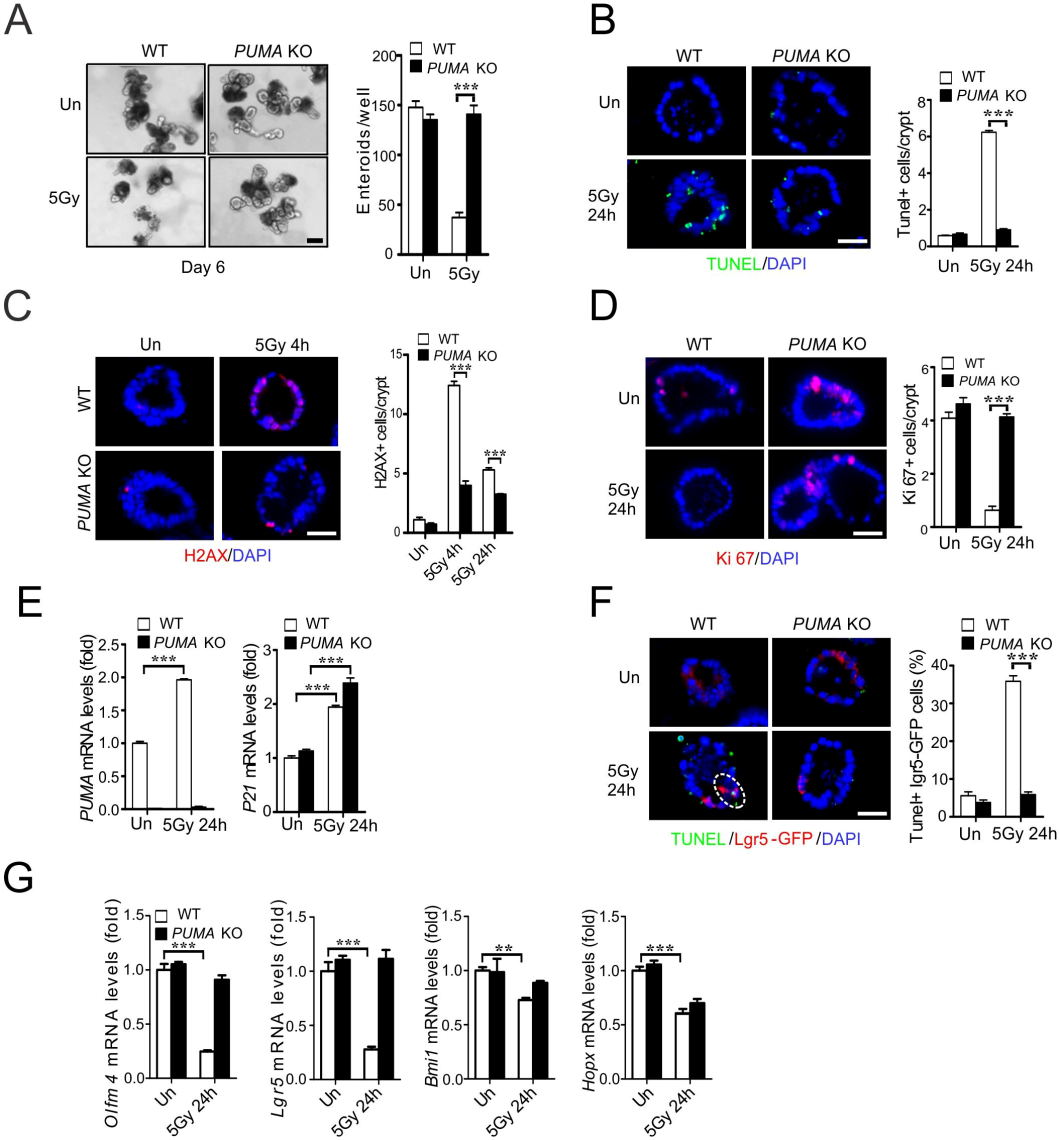
PUMA deficiency protects crypt culture and Lgr5+ cells against radiation by blocking apoptosis. Small intestinal crypts from WT and *PUMA* KO mice were plated in Matrigel and subjected to 5 Gy, or mock (Un) irradiation, 24 h after plating and harvested at the indicated time points. (A) Representative pictures of enteroids at day 6 and quantitation of enteroids with 5 or more buds. (B) Apoptosis was assessed by TUNEL staining at 24 h in WT and *PUMA* KO crypts. *Left*, representative pictures of TUNEL (green) staining. Right, quantitation of TUNEL index. (C) Representative pictures and quantitation of p-H2AX staining in cultured crypts at 4 and 24 h. (D) Representative pictures and quantitation of Ki 67 staining in cultured crypts at 24 h. (E) Expression of *PUMA* and *p21* transcripts was analyzed by real-time RT-PCR. (F) Crypts were isolated from *Lgr5-EGFP Cre-ER* background. Lgr5+ apoptosis was assessed by TUNEL/GFP staining at 24 h. *Left*, representative pictures of TUNEL (green), Lgr5 (red) and nuclei (DAPI) staining. *Right*, the percentage of Lgr5+ crypts containing one or more TUNEL+ cells. (G) Expression of *Olfm4*, *Lgr5*, *Bmi1* and *Hopx* transcripts was analyzed by real-time RT-PCR. Values are means ± SD, n = 3 wells from three different mice with each genotype. ***P < 0.001, **P < 0.01. Scale bar, A, 100 μm; B, C, D & F, 20 μm.

Our prior work indicated that *PUMA* KO mice show blocked apoptosis, and improved DNA repair and crypt regeneration through a p21-dependent mechanism^11^,^12^. To directly investigate if these effects are epithelial cell-intrinsic, we subjected cultured intestinal crypts isolated from WT or *PUMA* KO mice to ionizing irradiation. The radiation induced, dose-dependent suppression of growth and survival of WT enteroid culture was observed 6 days after 4–8 Gy (Figure S1A), which was significantly blocked in *PUMA* KO culture (Figure 1A and Figure S1A). TUNEL and active caspase-3 staining indicated that radiation induces marked apoptosis, which was blocked by over 80% in the *PUMA* KO group (Figure 1B and Figure S1B). We evaluated DNA damage and cell proliferation of irradiated crypt culture using markers such as p-H2AX, Ki67 and BrdU staining, and found reduced DNA double strand breaks and improved cell proliferation within 24 h in the *PUMA* KO group (Figure 1C, 1D and Figure S1C). Real-time PCR analysis showed a strong induction of *PUMA* and *p21* mRNA in the WT group 24 h after radiation, and a higher increase of *p21* in the *PUMA* KO group (Figure 1E).

PUMA induction by radiation is p53-dependent and preferentially in the intestinal stem and progenitor cells^11^. Using crypts from the *Lgr5-EGFP* mice^25^, we found that apoptosis in the Lgr5+ stem cells was strongly suppressed in *PUMA* KO crypts (Figure 1F and Figure S1B). Radiation rapidly reduced the expression of putative ISCs markers *Olfm4* and *Lgr5* at 24 h, which was blunted in *PUMA* KO crypts, while that of *Bmi1 or Hopx* was less sensitive to radiation or *PUMA* loss (Figure 1G). These results demonstrate radiation-induced crypt injury and Lgr5+ cell apoptosis is mediated via p53 and PUMA in a cell-intrinsic manner.

### CHIR99021 protects crypts and Lgr5+ cells against radiation in culture

**Figure 2:**
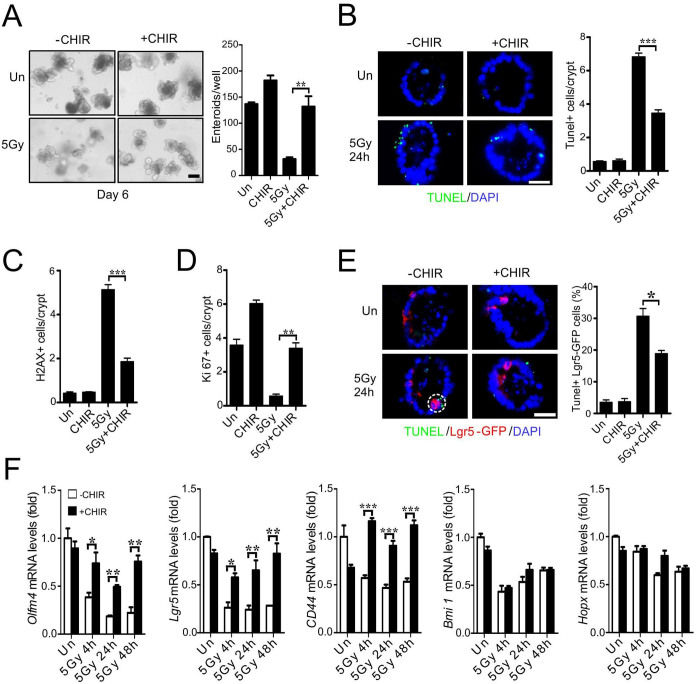
CHIR99021 protects crypt culture against radiation and blocks apoptosis. CHIR99021 (2.5 μM) was added to crypts 24 hr prior to 5 Gy or mock (Un) irradiation, and crypts were harvested at the indicated time points after radiation. (A) Representative pictures of enteroids at day 6 and quantitation of enteroids with 5 or more buds. (B) Apoptosis was assessed by TUNEL staining at 24 h. *Left*, representative pictures of TUNEL (green) staining. *Right*, quantitation of TUNEL index. (C) Quantitation of p-H2AX staining in cultured crypts 24 h after radiation. (D) Quantitation of Ki67 staining in cultured crypts 24 h after radiation. (E) Crypts were isolated from *Lgr5-EGFP Cre-ER* background. Apoptosis was assessed by TUNEL staining at 24 h. *Left*, representative pictures of TUNEL (green), Lgr5 (red) and nuclei (DAPI) staining. *Right*, quantitation of Lgr5+ crypts containing one or more TUNEL+ cells. (F) Expression of *Olfm4*, *Lgr5*, *CD44*, *Bmi* and *Hopx* transcripts was analyzed by real-time RT-PCR. Values are means ± SD. n = 3 wells from three different mice in each group. ***P < 0.001, **P < 0.01, *P < 0.05. Scale bar, A, 100 μm; B & E, 20 μm.

Using the crypt culture system, we screened nearly 20 compounds that have been shown to improve self-renewal or reprogramming in various systems, and identified GSK-3 inhibitor CHIR99021 as the most potent radiation protector. CHIR99021 treatment improved enteroid survival by over 3-fold six days after 5 Gy of radiation (Figure 2A). CHIR99021 significantly blocked apoptosis (Figure 2B and Figure S2A) and accumulation of p-H2AX positive cells (Figure 2C and Figure S2B), and enhanced proliferation at 24 h (Figure 2D and Figure S2C-D). CHIR99021 treatment also protected Lgr5+ cells against radiation-induced apoptosis (Figure 2E and Figure S2A), and prevented the decrease in *Olfm4*, *Lgr5* and *CD44*, but not *Bmi1* or *Hopx* (Figure 2F).

### CHIR99021 blocks radiation-induced PUMA induction in crypt culture through a p53-dependent mechanism

**Figure 3:**
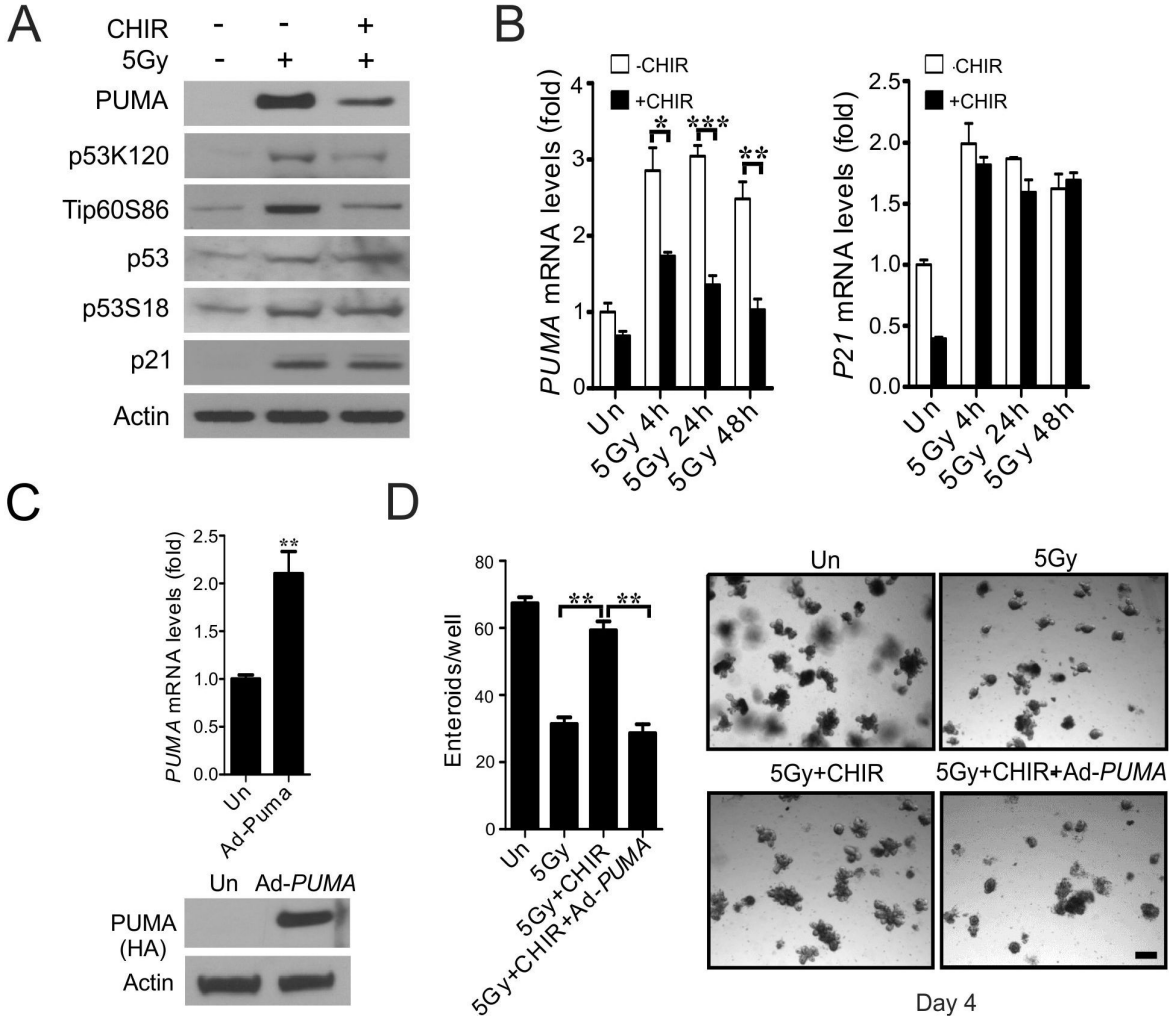
CHIR99021 inhibits p53-dependent PUMA induction and p53 K120-acetylation. Crypt culture were subjected to 5 Gy, or mock (Un) irradiation and harvested at indicated time points. (A) The expression of indicated proteins at 24 h in cultured crypts (3 wells pooled) was determined by western blotting. β-actin (Actin) was used as the control for loading. (B) Expression of *PUMA* and p21 transcripts was analyzed by real-time RT-PCR. (C) Cultured crypts were infected with an adenovirus encoding *PUMA* (Ad-PUMA) for 24 h, and *PUMA* mRNA levels and protein (HA) were analyzed by real-time RT-PCR and western blotting. (D) Cultured crypts were infected by Ad-PUMA at the same time with or without CHIR99021 treatment. *Left*, quantitation of enteroids with 5 or more buds at day 4. *Right*, representative pictures of enteroids at day 4. Scale bar, 100 μm. Values are means ± SD, n = 3 wells from three different mice in each group. ***P < 0.001, **P < 0.01, *P < 0.05.

We further investigated potential regulation of the p53 pathway by CHIR99021. Interestingly, CHIR99021 treatment did not affect p53 stabilization or phosphorylation (S18), but significantly blocked p53 acetylation (K120) (Figure 3A), which was suggested to be mediated by GSK-3-dependent phosphorylation (S86) and activation of Tip60^23^. Indeed, phosphorylation of Tip60 (S86) was strongly inhibited (Figure 3A). CHIR99021 treatment suppressed induction of PUMA, but not p21, at mRNA and protein levels (Figure 3A and 3B). Importantly, adenoviral expression of PUMA (Ad-PUMA)^26^ abrogated radioprotection by CHIR99021 (Figure 3C and 3D). Several growth factors such as bFGF1 and IGF1 have been shown to suppress radiation-induced crypt apoptosis by preventing p53 accumulation and PUMA induction in mice^1^,^27^. bFGF1 and IGF1 were found to significantly improve the survival of irradiated crypts in culture by blocking apoptosis and PUMA induction, which was associated with elevated phosphorylation of AKT and GSK-3β (S9, inhibitory) (Figure S2E-F, and data not shown). These results strongly suggest that CHIR99021 blocks p53-dependent induction of apoptosis and PUMA by preventing TIP60-mediated p53 acetylation.

### CHIR99021 protects mice and Lgr5+ cells against radiation-induced lethal GI injury

**Figure 4:**
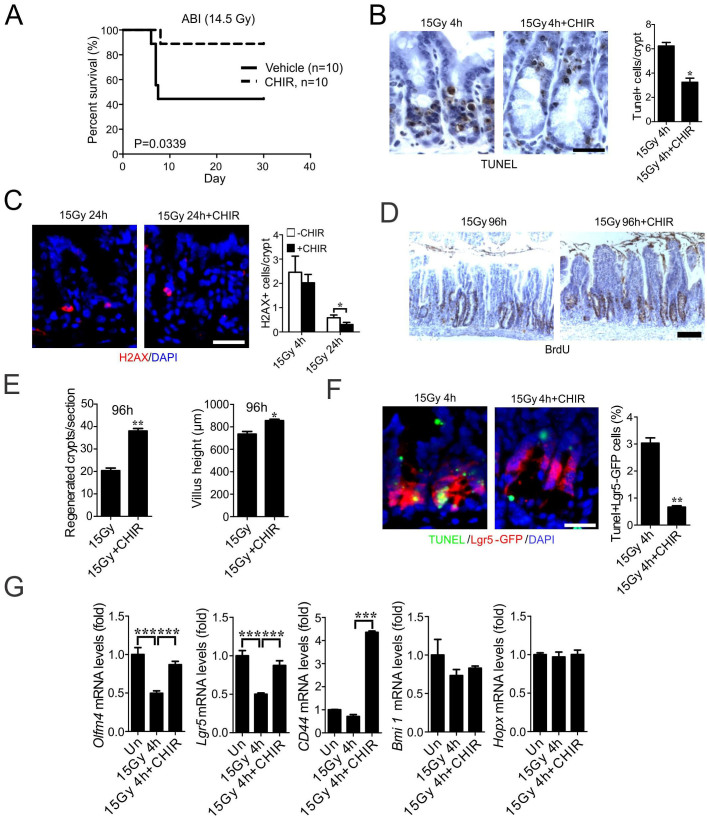
CHIR99021 protects mice from radiation-induced lethal GI injury. WT C57BL/6 mice were injected intraperitoneally with CHIR99021 (2 mg/kg) or vehicle (dimethyl sulfoxide) 4 h prior to 14.5 Gy ABI (A) or 15 TBI (B-G). (A) Survival curves of mice after ABI. n = 10 mice in each group. (B) Apoptosis in the intestinal crypts 4 h after TBI was assessed by TUNEL staining (brown). *Left*, representative pictures of TUNEL staining. *Right*, quantitation of TUNEL index. (C) Representative pictures and quantitation of p-H2AX staining in the intestinal crypts 4 h and 24 h after TBI. (D) Representative pictures of regenerated crypts identified by BrdU staining 96 h after TBI (brown). (E) Quantitation of regenerated crypts (left) and villus height (right) in D. (F) TUNEL staining in the crypts of *Lgr5-EGFP;Cre-ER* mice 4 h after TBI. *Left*, representative pictures of TUNEL (green), Lgr5 (red) and nuclei (DAPI) staining. *Right*, quantitation of Lgr5+ crypts containing one or more TUNEL+ cells. (G) Expression of *Olfm4*, *Lgr5*, *CD44*, *Bmi1* and *Hopx* transcripts was analyzed by real-time RT-PCR. (B-G), values are means ± SD, n = 3 mice in each group. ***P < 0.001, **P < 0.01, *P < 0.05. Scale bar, 25 μm.

Small molecules can be subjected to complex metabolism *in vivo*. Therefore, we determined the efficacy of CHIR99021 in mice. CHIR99021 (2 mg/kg) given once, 4 h before irradiation, significantly improved survival after 14.5 Gy abdominal irradiation (ABI) (Figure 4A). Using total body irradiation (TBI), we analyzed several well-characterized endpoints for radiation-induced GI injury and regeneration, including apoptosis at 4 h, DNA damage at 24 h, and crypt regeneration at 96 h. Consistent with our *in vitro* data, CHIR99021 treatment significantly blocked crypt apoptosis (Figure 4B and Figure S3A) and accumulation of p-H2AX+ cells (Figure 4C), and improved crypt regeneration and villus height (Figure 4D, 4E, Figure S3B). CHIR99021 treatment increased Lgr5+ cell survival by blocking apoptosis (Figure 4F, Figure S3C and S3D), and effectively prevented the reduction of *Olfm4*, *Lgr5* and *CD44* as early as 4 h (Figure 4G). The expression of *Bmi1* or *Hopx* was less sensitive to radiation or CHIR99021 treatment (Figure 4G). These results indicate CHIR99021 strongly protects mice, specifically Lgr5+ cells, in radiation-induced lethal GI injury *in vivo*.

### CHIR99021 inhibits p53-dependent induction of PUMA by irradiation in mice

**Figure 5:**
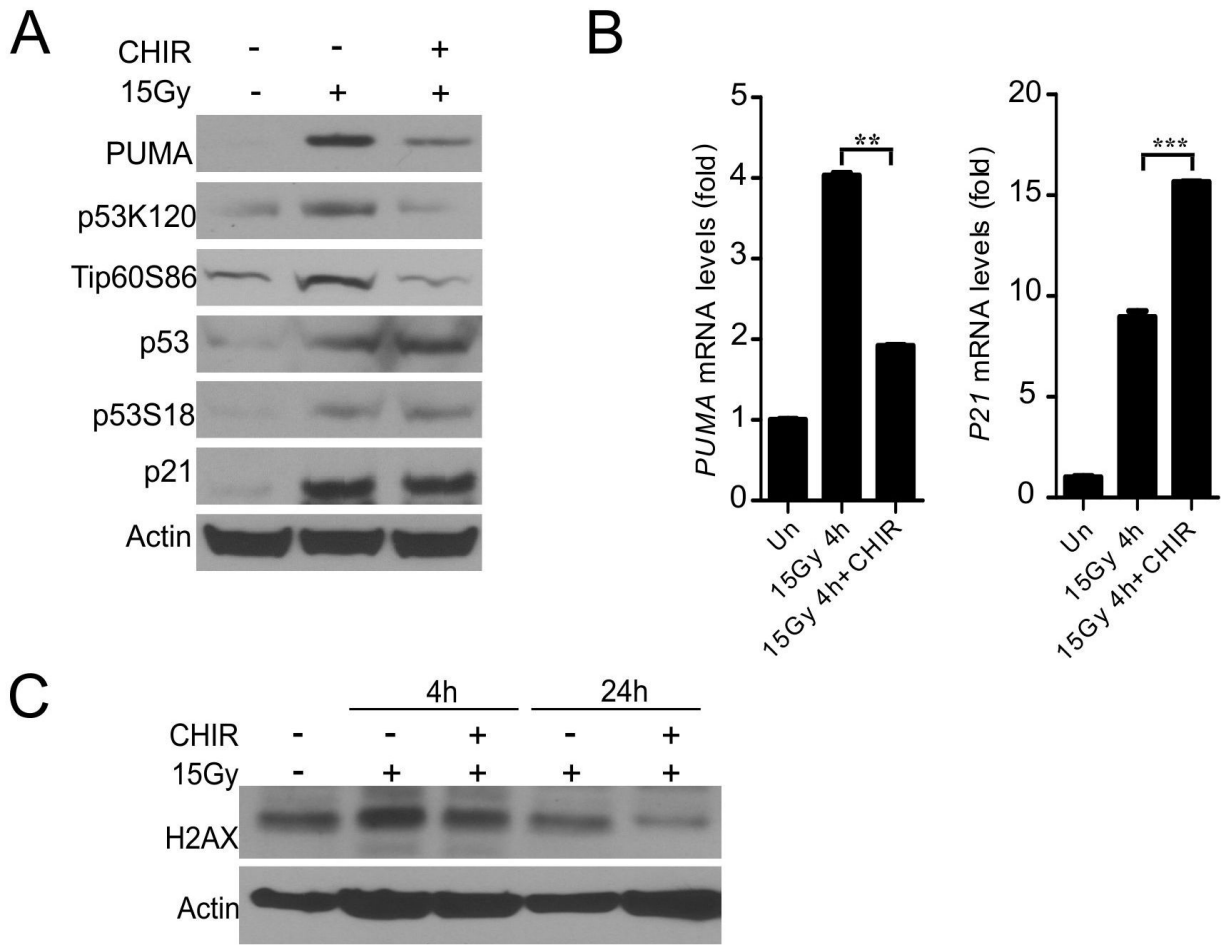
CHIR99021 suppresses induction of PUMA and p53 K120 acetylation by radiation in mice. Intestinal mucosa of WT mice were harvested after 15 Gy TBI with or without CHIR99021 treatment (2 mg/kg) 4 h prior to radiation. (A) The expression of indicated proteins at 4 h was analyzed by western blotting. β-actin was used as the control for loading. (B) The mRNA expression of *PUMA* and *p21* was analyzed by real-time RT-PCR. Values are means ± SD. n = 3 mice in each group. ***P < 0.001, **P < 0.01. (C) The expression of p-H2AX (pooled from 3 mice) was analyzed by western blotting. The lysates were pooled from 3 mice.

We then examined the effects of CHIR99021 treatment on the p53 pathway in the small intestinal mucosa. Consistent with the data from cultured crypts, CHIR99021 treatment blocked Tip60 phosphorylation, p53 acetylation and induction of PUMA, but not stabilization or phosphorylation of p53 or induction of p21 (Figure 5A and 5B). The levels of p-H2AX did not significantly differ in the control and CHIR99021 at 4 h, but significantly decreased at 24 h in CHIR99021-treated mice (Figure 5C). H2AX foci can result from DNA cleavage during apoptosis or death induction. Therefore, reduced H2AX foci in the CHIR or *PUMA* KO group likely reflect reduced overall cellular damage due to enhanced survival and some DNA repair indirectly. We additionally examined the levels of several Bcl-2 family proteins implicated in DNA damage-induced crypt apoptosis including Bax, Bak, Bcl-2, Bcl-xL or Mcl-1^1^, and did not observe significant changes upon CHRI99021 treatment (Figure S4). The GSK-3 inhibitor SB415286 was reported to suppress crypt apoptosis and Bax expression after 4 or 8 Gy TBI^28^, but failed to block apoptosis or PUMA induction in crypt culture or mice at the doses used in this study (Figure S5). These results indicate that CHIR99021 selectively ablates p53-dependent apoptosis and PUMA induction after radiation *in vivo*.

### CHIR99021 protects human intestinal stem cells against radiation injury by *PUMA* inhibition

**Figure 6:**
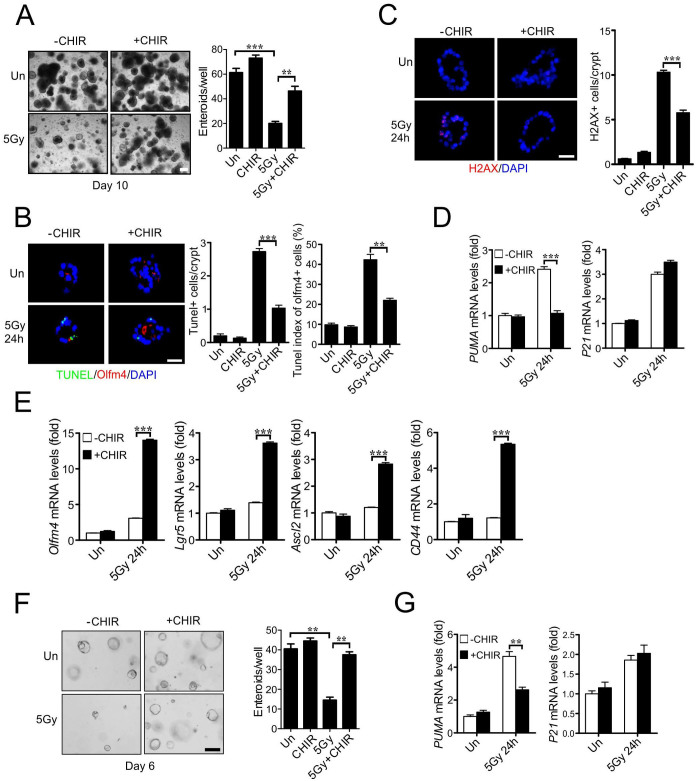
CHIR99021 protects human intestinal culture and stem cells against radiation injury. Two independent human intestinal cultures, EL1 (A-E) and MM1 (F-G), were plated and passaged in Matrigel as described in methods. CHIR99021 (2.5 μM) was added to culture medium 24 h before 5 Gy radiation, and cultures were harvested at indicated times after radiation. (A) Representative pictures of enteroids at day 10 and quantitation of enteroids ≥100 μm. (B) Apoptosis of was assessed by TUNEL staining at 24 h. Olfm4 labels putative stem cells. *Left*, representative pictures of TUNEL (green), Olfm4 (red) and nuclei (DAPI) staining. *Middle*, quantitation of TUNEL index. *Right*, quantitation of Olfm4+ cells containing one or more TUNEL+ cells. (C) Representative pictures (left) and quantitation of p-H2AX staining (right) 24 h after radiation. (D) Expression of *PUMA* and *p21* transcripts was analyzed by real-time RT-PCR. (E) Expression of *Olfm4*, *Lgr5*, *Ascl2* and *CD44* transcripts was analyzed by real-time RT-PCR. (F) Enteroid growth of MM1 culture was analyzed at day 6. (G) The expression of *PUMA* and *p21* transcripts in MM1 culture at 24 h was analyzed by RT-PCR. Values are means ± SD, n = 3 wells. ***P < 0.001, **P < 0.01. Scale bar, A and F, 200 μm; B and C, 30 μm.

It has not been possible to directly evaluate radiation response of normal human intestinal epithelial or stem cells. We decided to investigate the effects of CHIR99021 in human intestinal cultures established from two unrelated donors, which contained all differentiated epithelial lineages (Figure S6A). CHIR99021 was added to the intestinal culture (EL1) 24 h before 5 Gy radiation and found to significantly improve enteroid formation at 10 days (Figure 6A). CHIR99021 treatment significantly reduced apoptosis of the Olfm4+ cells, DNA damage, and improved cell proliferation as early as 24 h (Figure 6B and 6C, Figure S6B). CHIR99021 treatment blocked induction of PUMA, but not p21 (Figure 6D and Figure S6C), and significantly increased the expression of putative human ISC markers including *Olfm4*, *Lgr5*, *Ascl2* and *CD44* (Figure 6E). Similar results were obtained from an independent adult human intestinal culture with CHIR99021 treatment, including improved survival and growth of enteroids, suppression of *PUMA* expression and increased expression of ISC markers (Figure 6F and 6G, Figure S6D). Somewhat different from the patterns in mouse crypt culture, the levels of *Olfm* or *CD44*, but not *Lgr5*, decreased significantly within 24 h of radiation, while all four Wnt targets were highly induced by CHIR99021 treatment. This might reflect species-specific growth kinetics, Wnt responsiveness, a requirement for culture additives, or an inherent difficulty using Wnt targets as ISC markers during injury and regeneration. These results demonstrate that CHIR99021 protects human intestinal stem cells from radiation by blocking p53-dependent induction of apoptosis and PUMA.

## Discussion

There has been a growing interest to develop agents for the management of both short-term and long-term radiation injury in cancer patients, cancer survivors and victims of radiation disasters^2^,^3^,^8^,^9^. In this study, using crypt culture^24^,^29^, we demonstrate that p53-dependent apoptosis mediates radiation-induced acute ISC and crypt injury via a cell-intrinsic mechanism as suggested by *in vivo* studies^1^,^11^,^27^,^30^. We then identified CHIR99021 as a potent radiation protector of intestinal stem cells in mouse and human by selectively blocking p53-dependent induction of PUMA and apoptosis through Tip60-mediated posttranslational modification, without affecting the levels of p53 or p21 induction (Figure S6E).

Following DNA damage, p53 protein is stabilized and undergoes extensive modifications, and activating apoptosis in a tissue- and cell type-specific manner^31^,^32^. Our work is in agreement with the model that the modifications, not just levels of total protein, of p53 are responsible for radiation-induced apoptosis in human and mouse ISCs, which is likely to be mediated through the GSK-3-dependent Tip60 phosphorylation and p53 acetylation^20^,^21^,^22^,^23^ and inhibited by CHIR99021 (Figure S6E). This mechanism is distinct from preventing p53 accumulation by other GSK-3 inhibitor SB415286^18^ or growth factors^27^. Targeting p53 modifications might provide a novel way of combating p53-dependent and tissue-specific radiation toxicity. Since GSK-3 can be regulated by Wnt or NF-κB signaling^15^,^33^, it will be interesting to determine if suppression of apoptosis via the GSK-3/p53/PUMA axis is responsible for GI protection provided by R-spondin1 or Toll-like receptor (TLR) agonists (reviewed in Ref. 4). These studies might reveal potentially novel regulation of p53-mediated DNA damage response by Wnt or NF-κB signaling.

A major concern in normal tissue protection during cancer treatment is tumor cell protection. p53 or its function is lost in most human cancers and associated with therapeutic resistance^34^,^35^. Unlike *p53* KO mice, *PUMA* KO mice are highly resistant to radiation-induced lethal GI and bone marrow injury^11^,^36^,^37^. Additionally, *PUMA* KO mice show little or even reduced risk of cancer after radiation (as much as 28 Gy in 4 rounds of TBI)^38^,^39^ by preventing compensatory proliferation and clonal expansion of damaged cells. In addition, p53-dependent apoptosis might be compromised in p53 WT cancer due to frequent activation of the PI3K/AKT/mTOR or Ras/Raf/ERK pathways^40^,^41^. Thus, targeting p53-dependent apoptosis likely offers a high level of selectivity for radiation protection in normal tissue, but not p53-deficient cancer cells.

The critical cell populations in radiation-induced GI injury and regeneration are still heavily debated mainly due to lack of reliable markers^4^. Mounting evidence indicates Lgr5+ cells are critical for crypt regeneration after radiation while the CBCs are rapidly deleted during acute intestinal injury (this study)^7^,^42^,^43^,^44^. Our data on CHIR99021, *PUMA* KO and those on Wnt agonists including R-spondin1^45^,^46^,^47^ supports that Lgr5+ cell protection reduces GI injury and improves crypt regeneration in the setting of GI syndrome. We found that Lgr5+ cell protection is accompanied by maintained or increased expression of Wnt-responsive transcripts such as *Olfm4*, *Lgr5* and *CD44*, but much less so on *Bmi1* or *Hopx*. Lgr5+ cell protection and expansion might be beneficial for several reasons, not mutually exclusive, such as increased clonogenic cells and preventing the collapse of “the niche” to facilitate DNA repair. Since transcription is halted in cells undergoing apoptosis, it is plausible that increased cell survival preferentially elevates short-lived transcripts such as *Lgr5 or Olfm4*, over the long-lived ones. Characterization of radiation resistant ISCs will likely provide more insight.

In summary, we identified GSK-3 inhibitor CHIR99021 as an intestinal radioprotector in mice and humans by selectively blocking p53-dependent apoptosis and PUMA induction in intestinal stem and progenitor cells. These findings suggest a novel approach for radiation protection of normal intestinal stem cells, but not p53-deficient cancer cells, and suggest crypt culture as a useful model for studying cell-intrinsic DNA damage responses of ISCs.

## Methods

### Mice

The procedures for all animal experiments were approved by the Institutional Animal Care and Use Committee of the University of Pittsburgh. The procedures were carried out in "accordance" with the approved guidelines. Mice 6–10 weeks old were used. The *PUMA^+/+^* and *PUMA^−/−^* littermates on C57BL/6 background (F10)^48^ and *Lgr5-EGFP* (*Lgr5-EGFP-IRES-creERT2*) mice^25^ have been described. Mice were subjected to whole body irradiation (TBI)^11^, or abdominal irradiation (ABI) as described^30^. Mice were injected intraperitoneally (i.p.) with 2 mg/kg of CHIR99021 (Cat# C-6556, LC Laboratories, Woburn, MA) 4 h before radiation or 1 mg/kg of SB415286 (Cat# 1617, Tocris bioscience, Ellisville, MO) 28 h and 4 h before radiation. Mice were sacrificed to collect small intestines for histology analysis and western blotting. All mice were injected i.p. with 100 mg/kg of BrdU (Cat# 858811, Sigma-Aldrich) before sacrifice. Three or more mice were used in each group. More details are found in supplemental material.

### Small intestinal crypt and cell isolation, culture and treatment

Mouse crypts were isolated and cultured in Matrigel with a cocktail of factors as previously described in 24-well plates in crypt culture medium (Advanced DMEM/F12 containing 50 ng/ml EGF, 100 ng/ml Noggin, 500 ng/ml human R-spondin 1, 1 mM N-Acetylcysteine, 1% N2 supplement and B27 supplement)^24^,^42^. Other agents used include CHIR99021 (2.5 μM), SB415286 (2.5 μM), bFGF (400 ng/ml), IGF1 (100 ng/ml), Ad-PUMA (0.22 μl/well)^26^, which was added to the culture 24 h before radiation. Two human intestinal cultures were established from either single cell suspension or enriched crypt preparations with slightly different culture additives as detailed in supplemental materials. Similar results were obtained from at least three independent experiments using two or more donors (mice or human), and triplicate wells were included in each experiment. More details are found in supplemental material.

### Western blotting

Total protein was prepared from freshly isolated small intestine and cultured crypts, separately, and western blotting was performed as previously described^42^. Details on antibodies are found in supplemental material.

### Total RNA extraction and real-time reverse transcription PCR

Total RNA was isolated from tissues and cultured crypts using the Mini-RNA Isolation II Kit (Cat# R1055, Zymo Research) according to the manufacturer's protocol. Complementary DNA was generated using SuperScript III reverse transcriptase (Invitrogen). Real-time PCR was performed on CFX96 (Bio-Rad, Hercules, CA) with SYBR Green (Invitrogen, Carlsbad, CA)^11^,^42^. Detailed sequences for real-time PCR are found in supplementary tables (Table S1 and S2).

### Immunohistochemistry (IHC) and immunofluorescence (IF), TUNEL, BrdU staining, crypt microcolony assay

Slides were processed and stained as described^11^,^12^,^30^. Details are found in supplemental material.

### Statistical analysis

Statistical analysis was carried out using GraphPad Prism V software. The survival data were analyzed by log-rank test. Data were presented as means ± S.D. Statistical significance was calculated with unpaired student's *t*-test. *P* < 0.05 was considered to be significant. The means ± 1 S.D. are displayed in the figures where applicable. For crypt culture experiments, results of a representative experiment using 3 triplicate wells were shown.

## Supplementary Material

Supplementary Information Supplementary Information
