# Supplementary material for: Pharmacologically blocking p53-dependent apoptosis protects intestinal stem cells and mice from radiation
Source: Sci Rep. 2015 Apr 10;5:8566. doi: 10.1038/srep08566 (PMC4392360; doi:10.1038/srep08566)

Pharmacologically blocking p53-dependent apoptosis protects intestinal stem cells and mice from radiation

Xinwei Wang[1,2], Liang Wei[1,2], Julie M Cramer[1], Brian J. Leibowitz[1,2], Colleen Judge[1,2], Michael Epperly[2,3], Joel Greenberger[2,3], Fengchao Wang[4], Linheng Li[4], Matthias G. Stelzner[5], James C.Y. Dunn[6], Martin G. Martin[6] , Eric Lagasse[1], Lin Zhang[2,7], Jian Yu[1,2,3*]

[1]Department of Pathology, University of Pittsburgh School of Medicine, 5117 Centre Avenue, Pittsburgh, PA 15213

[2]University of Pittsburgh Cancer Institute, 5117 Centre Avenue, Pittsburgh, PA 15213

[3]Department of Radiation Oncology, University of Pittsburgh School of Medicine, 5117 Centre Avenue, Pittsburgh, PA 15213

[4]Department of Pathology, University of Kansas Medical Center, Stowers Institute for Medical Research, 1000 E 50th Street, Kansas City, MS 64110

[5] Department of Surgery, Veterans Affairs Greater Los Angeles Healthcare System, Los Angeles, CA 90073

[6]Departments of Surgery and Pediatrics, David Geffen School of Medicine, University of California, 10833 Le Conte Ave, Los Angeles, CA 90095

[7]Department of Pharmacology and Chemical Biology, University of Pittsburgh School of Medicine, 5117 Centre Avenue, Pittsburgh, PA 15213

*Correspondence:**

Jian Yu, Ph.D., Hillman Cancer Center Research Pavilion, Suite 2.26h, 5117 Centre Ave, Pittsburgh, PA 15213. Email: yuj2@upmc.edu; Phone: 412-623-7786; Fax: 412-623-7778

Supplemental Methods

Mice

The procedures for all animal experiments were approved by the Institutional Animal Care and Use Committee of the University of Pittsburgh. The methods were carried out in "accordance" with the approved guidelines. Mice 6-12 weeks old were used. The *PUMA*$^{+/+}$ and *PUMA*$^{-/-}$ littermates on C57BL/6 background (F10) were generated from heterozygote intercrosses. The previously described *Lgr5-EGFP* (*Lgr5-EGFP-IRES-creERT2*) mice [1] were crossed with *PUMA*$^{-/-}$ mice to generate *Lgr5-EGFP/PUMA*$^{+/-}$ mice. The *Lgr5-EGFP/PUMA*$^{+/+}$ and *Lgr5-EGFP/PUMA*$^{-/-}$ littermates were generated by *Lgr5-EGFP/PUMA*$^{+/-}$ intercrosses. Genotyping was performed as previously described for *PUMA* [2] and for *Lgr5* [1]. The mice were housed in micro-isolator cages in a room illuminated from 7:00 AM to 7:00 PM (12:12-hour light/dark cycle) and were allowed access to water and chow *ad libitum*.

For TBI models, mice were irradiated at dose 15 Gy at a rate of 76 cGy/min in a ^{137}Cs irradiator (Mark I; JL Shepherd and Associates, San Fernando, CA, USA). Mice were injected intraperitoneally (i.p.) with 2 mg/kg of CHIR99021 (Cat# C-6556, LC Laboratories, Woburn, MA) 4h before radiation or 1 mg/kg of SB415286 (Cat# 1617, Tocris bioscience, Ellisville, MO) 28h and 4h before radiation. Mice were sacrificed to collect small intestines for histology analysis and western blotting at 4, 24 and 96 h after radiation. All mice were injected i.p. with 100 mg/kg of BrdU (Cat# 858811, Sigma-Aldrich) before sacrifice. Three or more mice were used in each group.

Abdominal irradiation (ABI) was used for survival studies and administered in the form of X-ray with a clinical grade linear accelerator (Varian Medical Systems, Palo Alto, CA). For ABI

experiments, a 3-cm wide radiation band was used to deliver the required doses at a rate of 600 Monitor Units (146 cGy)/min to anesthetized animals in groups of 10-15 per run [3].

Small intestinal crypt and cell isolation, culture and treatment

The mouse crypts was isolated and cultured as previously described [4, 5]. Similar results were obtained from at least three independent experiments using two or more donor mice, and triplicate wells were included in each experiment. A total of 500 crypts were mixed with 50 µl Matrigel (Cat# 356231, BD Bioscience, Bedford, MA) and plated in 24-well plates. After gelling of Matrigel, 500 µl of crypt culture medium [Advanced DMEM/F12 (Cat# 12634-010, Invitrogen, Grand Island, NY) containing 50 ng/ml EGF (Cat# 315-09, Peprotech, Rocky Hill, NJ), 100 ng/ml Noggin (Cat# 250-38, Peprotech, Rocky Hill, NJ), 500 ng/ml R-spondin 1 (Cat#4645-RS, R&D Systems, Minneapolis, MN), 1 mM N-Acetylcysteine (Cat# A9165, Sigma-Aldrich, St. Louis, MO), 1% N2 supplement (Cat# 17502-048, Invitrogen, Grand Island, NY) and B27 supplement (Cat# 12587-010, Invitrogen, Grand Island, NY)] was added. 2.5 µM CHIR99021, 2.5 µM, SB415286, 400 ng/ml bFGF or 100 ng/ml IGF-1 [6] was added as needed. For passage, enteroids were removed from Matrigel and mechanically dissociated into single crypt domains and then transferred to fresh Matrigel. Passage was performed every 5 days with a 1:3 split ratio. Ad-PUMA-GFP [7] was added to matrigel mixtures (0.02 µl/ 50µl/), and medium (0.2 µl/500 ul) in 24-well culture plates (0.04 µl/mL) at the time of plating with or without CHIR9921. Crypts were irradiated at 5 Gy 24h after plating. Enteroid growth was quantified 6 days after radiation in primary culture and 4 days after radiation in passaged culture. The cultured crypts were harvested 4 h or 24 h after radiation, fixed with 10% formalin and mixed

with 2% agarose and then processed. Sections (5 μm) from paraffin-embedded crypt enteroids were subjected to immunostaining. Protein and RNA were isolated form cultured crypts following digestion of the Matrigel with Cell Recovery Solution (Cat# 354253, BD Bioscience, Bedford, MA).

For human fetal intestinal single cell isolation and culture, small intestines were obtained from a 23 week fetus from an elective abortion performed at Magee Women's Hospital, and collected by the University of Pittsburgh Health Sciences Tissue Bank, Pittsburgh, PA with approval from the University of Pittsburgh Institutional Review Board (IRB approval no. PRO10090385). Intestines were cut longitudinally in HBSS, contents rinsed, cut into 1-inch pieces, transferred to EBSS/1 mM EGTA/1% HEPES (Life Technologies, NY/Sigma-Aldrich, MO/Mediatech, VA) and minced. Tissue was then transferred to a tube and incubated for 5 min at room temperature. After an EBSS wash, the tissue was treated three times with a cocktail containing 1mg/mL collagenase II (Life Technologies, NY), 1mg/mL hyaluronidase (Sigma-Aldrich, MO), and 20 μg/mL DNase I (Roche, IN) in HBSS/1% HEPES for 20 min. Tissue/cell suspensions were passed through a 100 μm cell strainer (Fisher, PA) to isolate single cells from undigested tissue. Cells were plated on a monolayer of previously irradiated rodent epithelial feeder cells [8] at ~80,000 cells/cm^2 in DMEM/F12 supplemented with 0.5% FBS, 25 μg/mL gentamicin (Sigma-Aldrich, MO), 1% Insulin-Transferrin Selenium (ITS) (Mediatech, VA) and 0.1 ng/μL human R-Spondin2 (R&D, MN). When single cells were plated, 10 μM ROCK inhibitor, Y-27632 (Reagents Direct, CA), was added to the media for ~24 hours. Cultures were passaged at 2-3 weeks post-plating (~70% confluency) by incubating with EBSS/1 mM EGTA/1% HEPES followed by 0.25% Trypsin/0.1% EDTA. Expanded intestinal cells were suspended in 50μL

Growth Factor Reduced (GFR), phenol-red free Matrigel™ (BD Bioscience, CA) and plated in tissue culture dishes at low density (~50,000 cells/well of 48-well plate). After solidification at 37°C for 30 min, 500µL of media was added to each well (See JMC Table). For the first 24 hours, the media included Y-27632 to prevent anoikis. Subsequently, cultured cells were treated with 2.5 µM CHIR99021 3 days after plating in Matrigel and irradiated at 5 Gy 24h after CHIR99021 treatment. Enteroid growth was quantified 10 days after radiation. The cells cultured in Matrigel were harvested 24 h after radiation, fixed with 10% formalin and mixed with 2% agarose and then processed. Sections (5 µm) from paraffin-embedded enteroids were subjected to immunostaining. Protein and RNA were isolated form cultured cells following digestion of the Matrigel with Cell Recovery Solution 24 h after radiation.

Human adult intestinal crypts were isolated as previously described [9]. Fresh tissues were obtained with appropriate IRB approval from the UCLA Department of Pathology Translational Pathology Core Laboratory. Isolated crypts were mixed with 50 ul Matrigel and plated in 24-well plates. Culture medium included 50% conditioned media from cultured myofibroblast and 50% Advanced DMEM/F12 supplemented with 2 mM GlutaMax, 10 mM Hepes, 1 mM N-Acetylcysteine, 50 ng/ml EGF, 100 ng/ml Noggin, 1 µg/ml R-spondin 1,100 ng/ml FGF10, 10 uM Y27632, 1% N2 supplement and B27 supplement [9]. Passage was performed every 4 days with a 1:3 split ratio. 2.5 µM CHIR99021 was added as needed. One day after plating, crypts were irradiated at 5 Gy. Enteroid growth was quantified 6 days after radiation. RNA was isolated from cultured crypts following digestion of the Matrigel with Cell Recovery Solution 24h after radiation.

Western blotting

Total protein was prepared from freshly isolated small intestine [5] and cultured crypts, separately, western blotting was performed as previously described [5]. Primary antibodies included PUMA (mouse) (Cat# ab9643, Abcam, Cambridge, MA), PUMA (human specific) [7], p53 (acetyl K120) (Cat# ab78316, Abcam, Cambridge, MA), Tip60 (phospho S86) (Cat# ab73207, Abcam, Cambridge, MA), p53 (Cat# sc-6243, Santa Cruz, Santa Cruz, CA), p53 (ser20, human) (S18, mouse) (Cat# 9287, Cell signaling, Danvers, MA), p21 (Cat# sc-397, Santa Cruz, Santa Cruz, CA), Bak (Cat# 06-536, Upstate, Billerica, MA), Bax (Cat# sc-493, Santa Cruz, Santa Cruz, CA), Bcl-xL (Cat# 610212, BD Biosciences, Bedford, MA), Bcl-2 (Cat# M0887, Dako, Carpenteria, CA), Mcl-1 (Cat# 559027, BD Biosciences, Bedford, MA), p-H2AX (Cat# 07-164, Upstate, Billerica, MA), AKT (Cat# 9272, Cell signaling, Danvers, MA), p-AKT (Cat# 4058, Cell signaling, Danvers, MA), p-GSK3β (S9) (Cat# 8566, Cell signaling, Danvers, MA) and β-actin (Cat# 5441, Sigma, St. Louis, MO).

Total RNA extraction and real-time reverse transcription PCR

Total RNA was isolated from tissues and cultured crypts using the Mini-RNA Isolation II Kit (Cat# R1055, Zymo Research) according to the manufacturer’s protocol. Complementary DNA was generated using SuperScript III reverse transcriptase (Invitrogen). Real-time PCR was performed on CFX96 (Bio-Rad, Hercules, CA) with SYBR Green (Invitrogen, Carlsbad, CA). Detailed sequences for real-time PCR are found in supplementary tables (Table S1 and S2).

Immunohistochemistry (IHC) and immunofluorescence (IF)

Slides were deparaffinized, rehydrated, and treated 3% hydrogen peroxide. Antigen retrieval was performed by boiling the sections for 10 minutes in 0.1 M Citrate Buffer Antigen Retrieval Solution (pH 6.0). Nonspecific antibody binding was blocked using 15% goat serum for 30 minutes. For active caspase 3 IHC, the slides were then incubated with rabbit polyclonal anti-caspase 3 antibody (Cat# 9661, Cell signaling, Danvers, MA) at 1:25 dilution at $4^{o}C$ overnight. The signals were detected with the ABC kit and DAB kit (Vector Laboratories, Burlingame, CA). The sections were counter-stained with hematoxylin. For active caspase 3, γ-H2AX, Ki67, GFP, Olfm4, Lysozyme, Muc2, Chromogranin A IF, the slides were incubated with rabbit polyclonal anti-caspase 3 antibody at 1:25 dilution, mouse monoclonal anti-p-H2AX antibody (Cat# 05-636, Millipore, Billerica, MA) at 1:100 dilution, rat monoclonal anti-Ki67 antibody (Cat# M7249, DAKO, Carpinteria, CA) at 1:100 dilution, mouse monoclonal anti-GFP antibody (Cat# sc-9996, Santa Cruz, Santa Cruz, CA) at 1:50 dilution, mouse monoclonal anti-Olfm4 antibody (Cat# N212, from Dr. Yasui) at 1:50 dilution [10], goat monoclonal anti-Lysozyme antibody (Cat# sc-27958, Santa Cruz, Santa Cruz, CA) at 1:50 dilution, rabbit monoclonal anti-Muc2 antibody (Cat# sc-15334, Santa Cruz, Santa Cruz, CA) at 1:100 dilution, rabbit monoclonal anti-Chromogranin A antibody (Cat# ab15160, Abcam, Cambridge, MA) at 1:50 dilution, respectively. Antibody-antigen complexes were visualized by incubation with Alexa Fluor 594 (Invitrogen, Carlsbad, CA) and counterstained with DAPI (Vector Laboratories, Burlingame, CA). For TUNLE/GFP, TUNEL/active caspase 3 and TUNEL/Olfm4 double staining, TUNEL staining was performed following GFP, active caspase 3 or Olfm4 staining. Cells with positive staining were scored in at least 100 crypts per mouse or 30 crypts per cultured crypts block and reported as means ± SD. Three mice were used in each group.

TUNEL, BrdU staining and crypt microcolony assay

TUNEL (Terminal deoxynucleotidyl transferase mediated dUPT nick end labeling) staining was conducted as described [11]. In brief, TUNEL staining was conducted with the ApopTag Peroxidase Kit or ApopTag Flurescein In Situ Apoptosi Detection Kit (Cat# S7101 or S7110, Chemicon International, Temecula, CA). TUNEL positive cells were scored in 100 crypts per mouse or 30 crypts per cultured crypts block and reported as means ± SD. Three mice were used in each group.

For BrdU IHC, sections were deparaffinized and treated with proteinase K (20 μg/ml) for 15 min at 37°C and 2N HCl for 60 min. Staining was carried out following a standard peroxidase protocol with an anti-BrdU antibody (Cat# A21301MP, Invitrogen) at 1:100 dilution overnight at 4°C, secondary goat-anti-mouse-biotin antibody (Cat# 31802, Pierce) at 1:100 dilution, then amplified with the VectaStain ABC kit and developed by DAB. For BrdU IF, following incubation with biotinylated primary antibody, an Alexa Fluor-594 secondary antibody (Cat# A11005, Invitrogen, Carlsbad, CA) was used for detection, and DAPI was used for counterstaining and mounting.

The crypt microcolony assay was used to quantify stem cell survival by counting regenerated crypts in BrdU-stained cross sections 4 days after radiation as described previously [11] (15). The regenerated crypts contained 5 or more BrdU positive cells with a lumen. At least three mice were used in each group and the data from BrdU staining was reported as means ± SD.

Supplemental Table

Table S1: Mouse primers used for RT-PCR analysis		
**Gene**	**Primer**	**Sequence**
PUMA	Forward	5′-ATGGCGGACGACCTCAAC-3′
	Reverse	5′-AGTCCCATGAAGAGATTGTACATGAC-3′
p21	Forward	5′-ATGTCCAATCCTGGTGATGT-3′
	Reverse	5′-TGCAGCAGGGCAGAGGAAGT-3′
Olfm4	Forward	5′-GCCACTTTCCAATTTCAC-3′
	Reverse	5′-GAGCCTCTTCTCATACAC-3′
Lgr5	Forward	5′-GACAATGCTCTCACAGAC-3′
	Reverse	5′-GGAGTGGATTCTATTATTATGG-3′
CD44	Forward	5′- CCCCCTTTCTTTTTCCAGTT-3′
	Reverse	5′-ACTTTCTGCCCCTCTCCACT-3′
Bmi1	Forward	5′-TATAACTGATGATGAGATAATAAGC-3′
	Reverse	5′-CTGGAAAGTATTGGGTATGTC-3′
Hopx	Forward	5′-CACCACGCTGTGCCTCATCG-3′
	Reverse	5′-CAAAACAGCCTGGGTAAGCC-3′
GAPDH	Forward	5′-CTCTGGAAAGCTGTGGCGTGATG-3′
	Reverse	5′-ATGCCAGTGAGCTTCCCGTTCAG-3′

Table S2: Human primers used for RT-PCR analysis		
**Gene**	**Primer**	**Sequence**
PUMA	Forward	5′-CGACCTCAACGCACAGTACGA-3′
	Reverse	5′- AGGCACCTAATTGGGCTCCAT-3′
p21	Forward	5′-AGCGGAACAAGGAGTCAG -3′
	Reverse	5′-CGTTAGTGCCAGGAAAGAC -3′
Olfm4	Forward	5′-CTGCCAGACACCACCTTTCC-3′
	Reverse	5′-CTCGAAGTCCAGTTCAGTGTAAG-3′
Lgr5	Forward	5′-AACAGTCCTGTGACTCAACTCAAG-3′
	Reverse	5′-TTAGAGACATGGGACAAATGCCAC-3′
Ascl2	Forward	5′-CGTGAAGCTGGTGAACTTGG-3′
	Reverse	5′-GGATGTACTCCACGGCTGAG-3′
CD44	Forward	5′-GACAAGTTTTGGTGGCACG -3′
	Reverse	5′-CACGTGGAATACACCTGCAA -3′
β-actin	Forward	5′-GACCTCACAGACTACCTCAT -3′
	Reverse	5′-AGACAGCACTGTGTTGGCTA-3′

Supplemental References

1. Barker N*, et al.* Identification of stem cells in small intestine and colon by marker gene Lgr5. *Nature* **449**, 1003-1007 (2007).

2. Wu B*, et al.* p53 independent induction of PUMA mediates intestinal apoptosis in response to ischaemia-reperfusion. *Gut* **56**, 645-654 (2007).

3. Leibowitz BJ*, et al.* Ionizing irradiation induces acute haematopoietic syndrome and gastrointestinal syndrome independently in mice. *Nat Commun* **5**, 3494 (2014).

4. Sato T*, et al.* Single Lgr5 stem cells build crypt-villus structures in vitro without a mesenchymal niche. *Nature* **459**, 262-265 (2009).

5. Qiu W*, et al.* ADAR1 is essential for intestinal homeostasis and stem cell maintenance. *Cell Death Dis* **4**, e599 (2013).

6. Qiu W, Leibowitz B, Zhang L, Yu J. Growth factors protect intestinal stem cells from radiation-induced apoptosis by suppressing PUMA through the PI3K/AKT/p53 axis. *Oncogene* **29**, 1622-1632 (2010).

7. Yu J, Wang Z, Kinzler KW, Vogelstein B, Zhang L. PUMA mediates the apoptotic response to p53 in colorectal cancer cells. *Proc Natl Acad Sci U S A* **100**, 1931-1936 (2003).

8. Ehmann UK, Peterson WD, Jr., Misfeldt DS. To grow mouse mammary epithelial cells in culture. *J Cell Biol* **98**, 1026-1032 (1984).

9. Lahar N*, et al.* Intestinal subepithelial myofibroblasts support in vitro and in vivo growth of human small intestinal epithelium. *PLoS One* **6**, e26898 (2011).

10. Qiu W*, et al.* Chemoprevention by nonsteroidal anti-inflammatory drugs eliminates oncogenic intestinal stem cells via SMAC-dependent apoptosis. *Proc Natl Acad Sci U S A* **107**, 20027-20032 (2010).

11. Qiu W*, et al.* PUMA regulates intestinal progenitor cell radiosensitivity and gastrointestinal syndrome. *Cell Stem Cell* **2**, 576-583 (2008).

Supplemental Figure Legends

Figure S1. PUMA deficiency suppresses radiation-induced apoptosis and enhances regeneration of cultured crypts. Crypt culture was subjected to the indicated doses (0-8 Gy) of

irradiation and harvested at the indicated time points. (A) Quantitation of enteroids with 5 or more buds at day 6 following increased doses of radiation. (B) Apoptosis was assessed by active caspase 3 staining 24h after 5 Gy radiation, *Lgr5-EGFP Cre-ER* background. *Left*, representative pictures of active caspase 3 (red), Lgr5 (green) and nuclei (DAPI) staining. Quantitation of Lgr5+ crypts containing one or more active caspase 3+ cells (right). (C) Representative pictures and quantitation of BrdU staining at 24h. Scale bar, B, 50 µm; C, 20 µm. Values are means ± SD, n = 3 wells. ***P<0.001.

Figure S2. CHIR99021 suppresses radiation-induced apoptosis and enhances regeneration of cultured crypts. Crypt culture was subjected to 5 Gy or mock (Un) irradiation with or without CHIR99021 or growth factor treatment, and harvested at the indicated time points. (A) Active caspase 3 staining (red) at 24h in *Lgr5-EGFP Cre-ER* background. *Left*, representative pictures of active caspase 3 (red), Lgr5 (green) and nuclei (DAPI) staining. *Middle*, quantitation of active caspase 3 staining. *Right*, quantitation of Lgr5+ crypts containing one or more active caspase 3+ cells. (B) Representative pictures of p-H2AX staining at 24h. (C) Representative pictures of Ki67 staining at 24h. (D) Representative pictures and quantitation of BrdU staining at 24h. (E) Representative pictures of enteroids at day 4 following radiation with 5 or more buds and quantitation. bFGF (400ng/ml) or IGF1 (100 ng/ml) was added at plating. Values are means ± SD. n = 3 wells from three different mice in each group. ***P<0.001, **P<0.01. Scale bar, A, 50 µm; B, C & D, 20 µm; E, 100 µm. (F) The expression of the indicated proteins in culture treated as in (E) 4 and 24h after radiation (pooled from 3 wells) was determined by western blotting. β-actin was used as the control for loading.

Figure S3. CHIR99021 protects crypts and Lgr5+ cells against radiation injury in mice. C57BL/6 mice were injected intraperitoneally with CHIR99021 (2 mg/kg) or vehicle (dimethyl sulfoxide) 4h prior to 15 Gy TBI, and intestinal tissues were harvested at indicated times after 15 Gy TBI. (A) Apoptosis in the intestinal crypts of WT at 4h was assessed by active caspase 3 staining (brown). *Left*, representative pictures of active caspase 3 staining. *Right*, quantitation of active caspase 3 staining. (B) Representative pictures of regenerated crypts identified by BrdU staining (brown) 96h after 15 Gy TBI. (C) Active caspase 3 staining in *Lgr5-EGFP Cre-ER* at 4h. *Left*, representative pictures of active caspase 3 (red), Lgr5 (green) and nuclei (DAPI) staining 4h after TBI. *Right*, quantitation of Lgr5+ crypts containing one or more active caspase 3+ cells. (D) Quantitation of Lgr5+ cells per cross intestinal section at 4h and 24h. Values are means ± SD. n = 3 mice in each group. *$P<0.05$. Scale bar, 50 µm.

Figure S4. CHIR99021 treatment does not affect the expressions of Bcl-2 family members in the intestine of irradiated mice. Intestinal mucosa of WT mice were harvested 4h after 15 Gy TBI with or without treatment of CHIR99021 (2 mg/kg). The expression of indicated proteins (pooled from 3 mice) was determined by western blotting. β-actin was used as the control for loading.

Figure S5. SB415286 does not protect against radiation-induced crypt injury in culture or in mice. (A) The effects of SB415286 (2.5 µM) on enteroid formation 6 days after 5 Gy irradiation. *Left*, representative pictures. *Right*, quantitation of enteroids with 5 or more buds.

Values were means ± SD, n = 3 wells. (B) Mice were subjected to 15 Gy TBI with or without SB treatment (1 mg/kg). Apoptosis in the intestinal crypts of WT mice at 4h was assessed by TUNEL staining. *Left*, representative pictures of TUNEL staining. *Right*, quantitation of TUNEL staining. (C) Mice were treated as in (B). Representative pictures of regenerated crypts identified by BrdU staining (brown) 96h. (B) and (C), values are means ± SD. n = 3 mice in each group. Scale bar, A, 100 µm; B & C, 50 µm.

Figure S6. CHIR99021 protects human intestinal culture against radiation injury. Human intestinal cultures, EL1 (A-C) and MM1 (D) were treated as in Figure 6, and harvested at indicated time points after 5 Gy irradiation. (A) Differentiation in unirradiated human intestinal culture indicated by immunofluorescence staining for Lysozyme (red, Paneth cells), Muc2 (red, goblet cells) and Chromogranin A (red, enteroendocrine cells). Counterstain, DAPI (blue). (B) Representative pictures and quantitation of Ki 67 staining 24h after radiation. (C) The expression of PUMA and p21 (3 wells pooled) was determined by western blotting. * IR inducible PUMA isoform. β-actin was used as the control for loading. (D) Expression of *Olfm4*, *Lgr5, Ascl2* and *CD44* transcripts in MM1 culture 24 hr after radiation. (B) and (D) values are means ± SD, n = 3 wells from a representative experiment. ***P<0.001. **P<0.01. Scale bar, 30 µm. (E) A model. GSK3 inhibition selectively blocks p53-dependent induction of apoptosis and PUMA (destructive) while it maintains induction of p21 and DNA repair (protective).

Wang *et al. Supple 1*

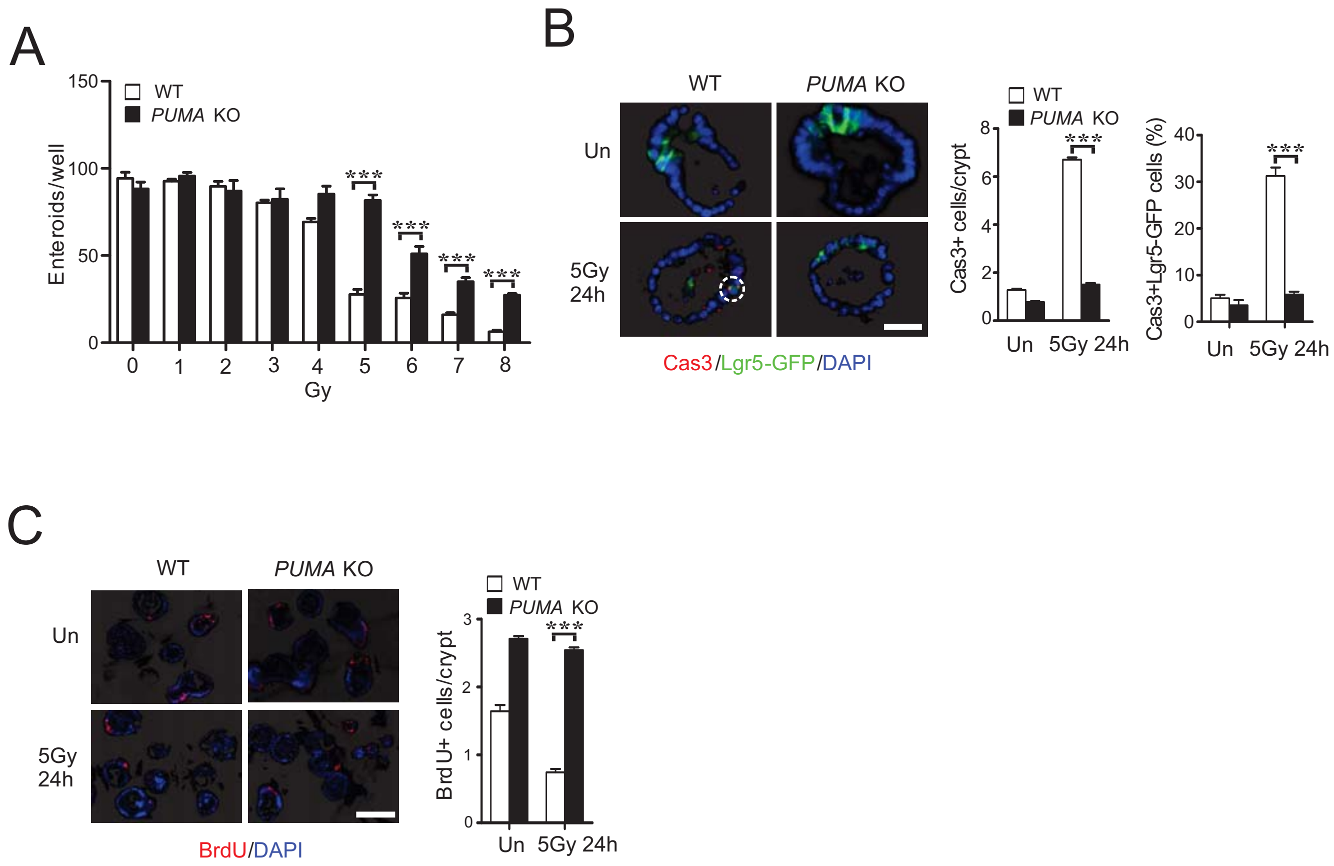

Wang *et al. Supple 2*

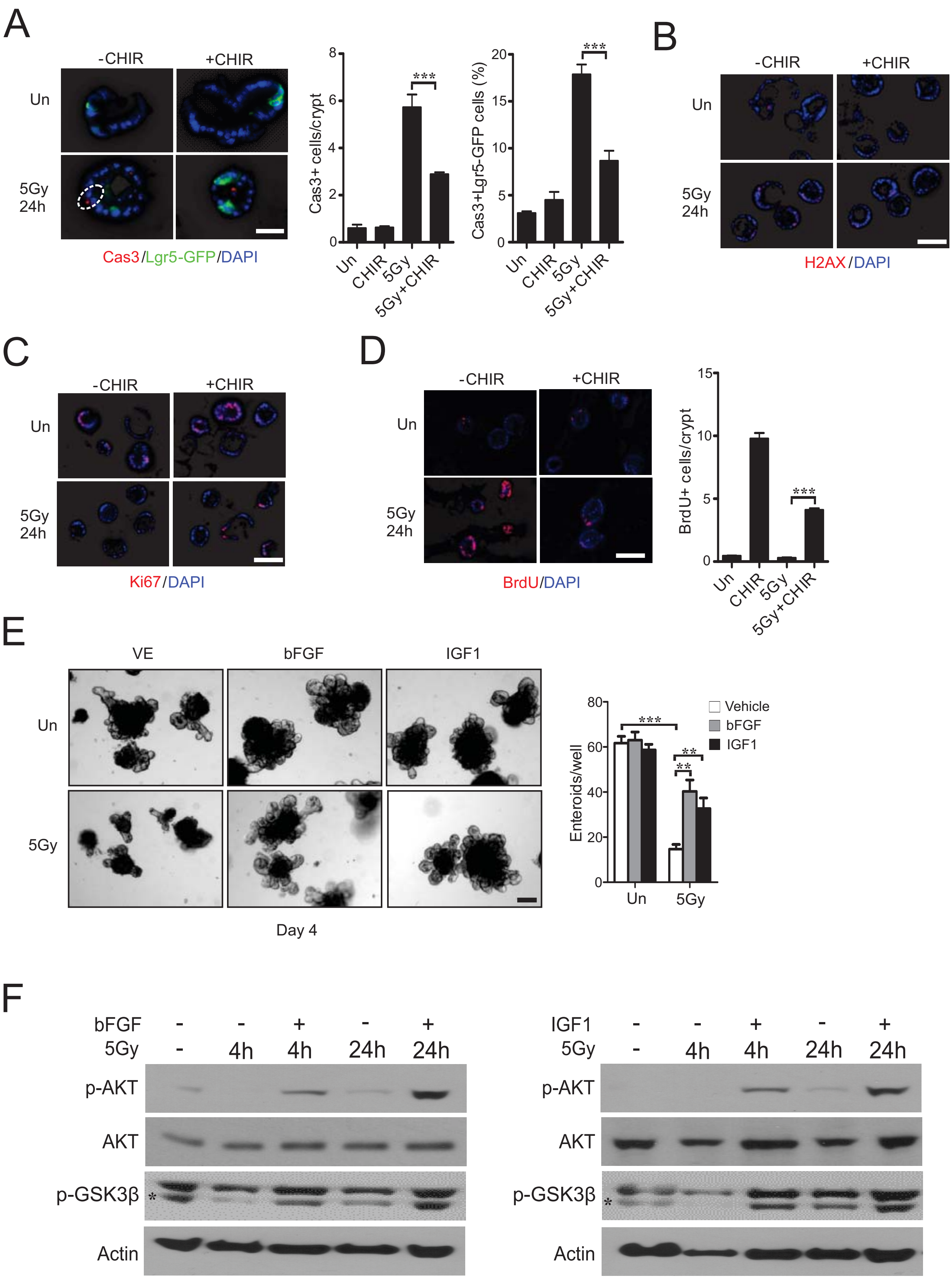

Wang *et al. Supple 3*

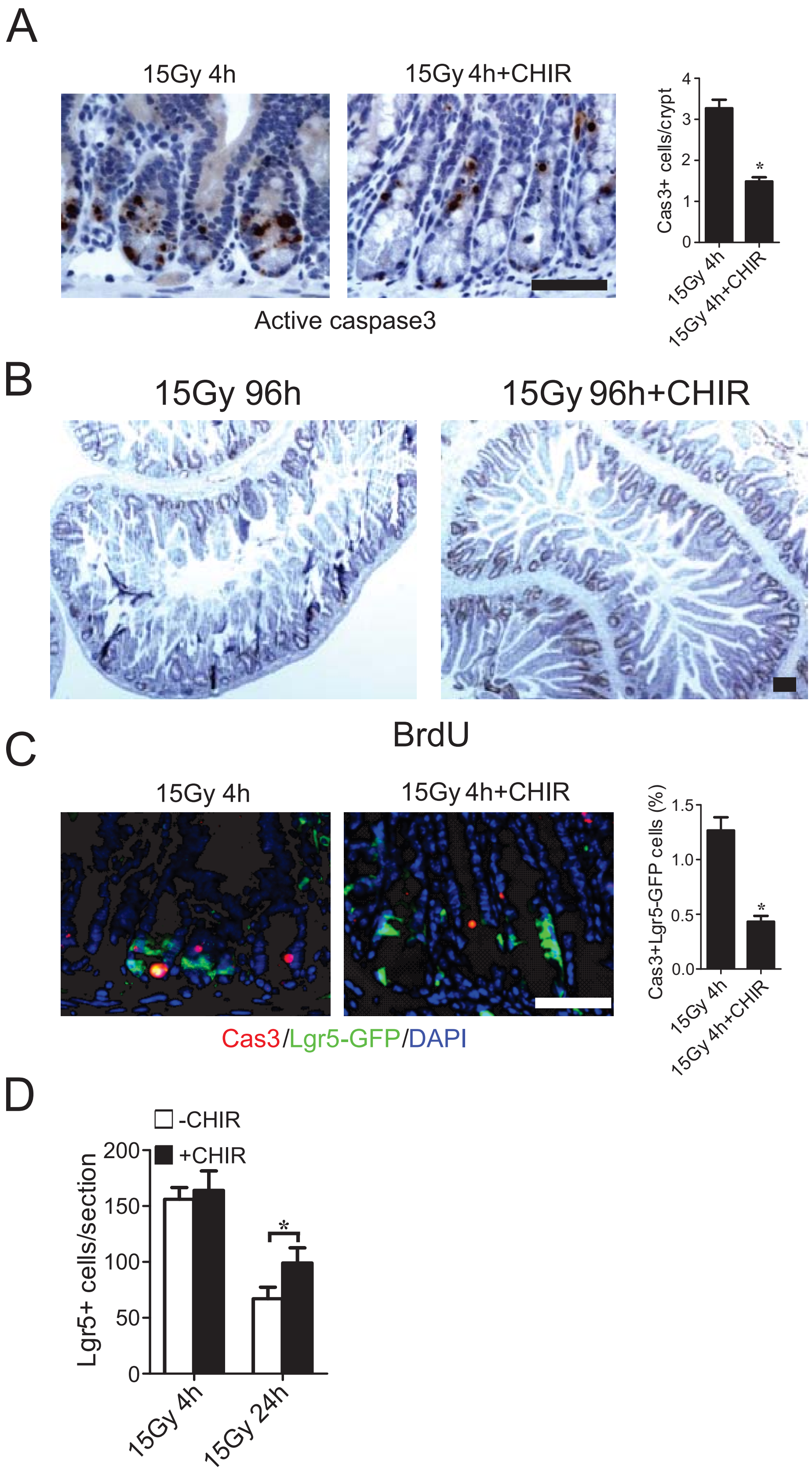

Wang *et al. Supple 4*

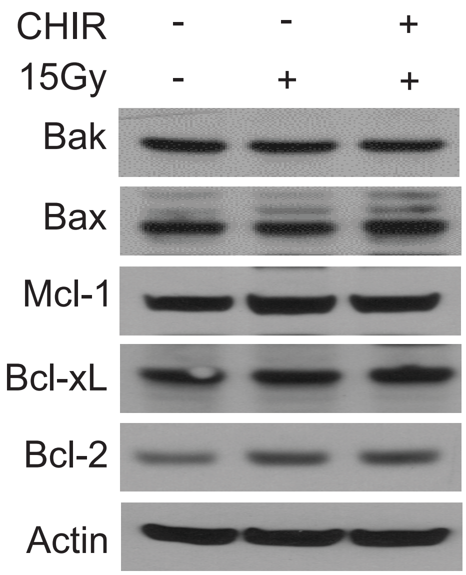

Wang *et al. Supple 5*

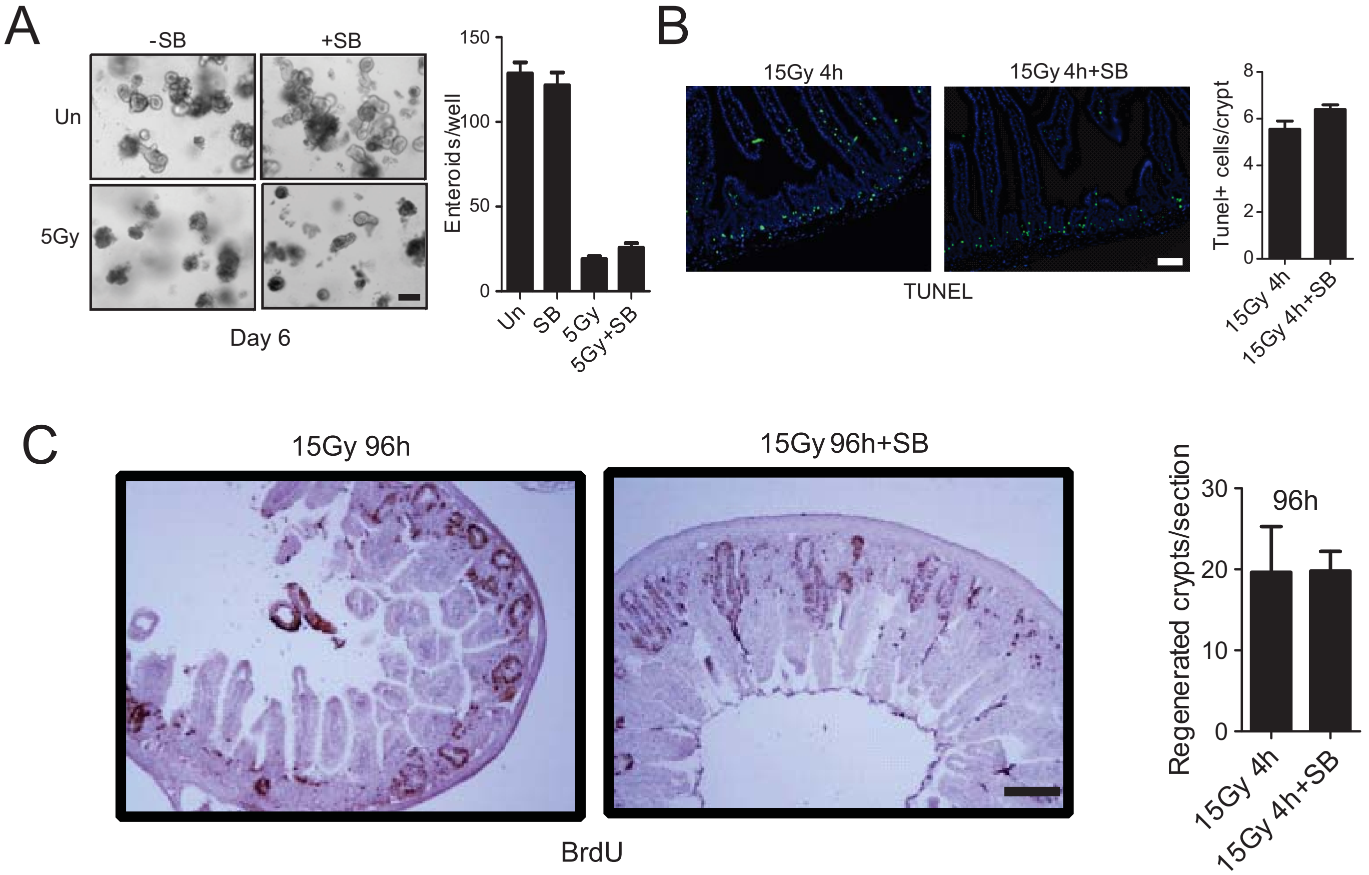

Wang *et al. Supple 6*

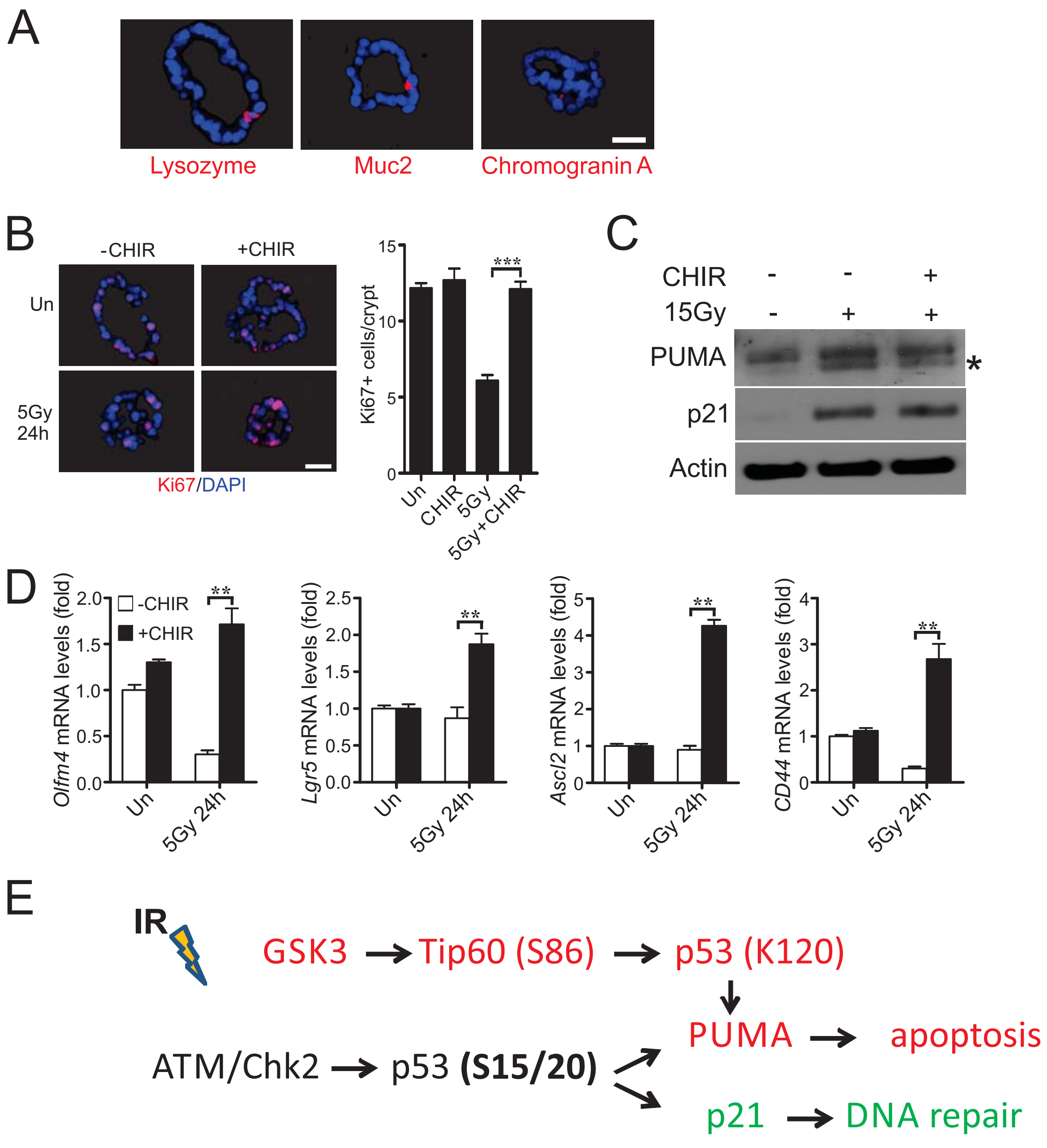

Supplement: Supplementary Information [file srep08566-s1.pdf]
